# Glasgow prognostic score predicts prognosis of non-small cell lung cancer: a meta-analysis

**DOI:** 10.1186/s40064-016-2093-9

**Published:** 2016-04-12

**Authors:** Lucheng Zhu, Sumei Chen, Shenglin Ma, Shirong Zhang

**Affiliations:** Affiliated Hangzhou Hospital of Nanjing Medical University, No. 261, Huansha Road, Shangcheng District, Hangzhou, 310006 People’s Republic of China; Hangzhou First People’s Hospital, No. 261, Huansha Road, Shangcheng District, Hangzhou, 310006 People’s Republic of China; Affiliated Hangzhou First People’s Hospital of Zhejiang Chinese Medical University, Hangzhou, 310006 People’s Republic of China; Hangzhou Cancer Hospital, Hangzhou, 310006 People’s Republic of China

**Keywords:** Glasgow prognostic score, GPS, Non-small cell lung cancer, Prognosis, Meta-analysis

## Abstract

**Objective:**

Glasgow prognostic score (GPS), an inflammation-based scoring system, has been evaluated in various cancers. However, its clinical significance remains unclear in non-small cell lung cancer (NSCLC). Therefore, it is necessary to conduct a meta-analysis to explore the prognostic value of GPS in NSCLC patients.

**Methods:**

A quantitative meta-analysis was performed through a systematic search in PubMed, Web of Science, and the Cochrane Library. The pooled hazard ratios (HRs) of overall survival (OS) were calculated and compared.

**Results:**

A total of 12 studies comprising 2669 patients were included in this meta-analysis. Compared with GPS 0 group, patients in GPS 1–2 group exhibited a reduced OS with a pooled HR of 1.89 (95 % CI 1.57–2.27, *p* < 0.001; *I*^2^ = 54 %). Six studies had sufficient data to calculate HRs of OS for GPS 1 and GPS 2 groups. Analysis revealed that GPS 2 group had a statistically significant reduced OS compared with GPS 1 group with a pooled HR of 1.87 (95 % CI 1.18–2.97, *p* = 0.008; *I*^2^ = 72 %). Study type (retrospective vs. prospective) and disease stage could partially explain the heterogeneity of each study by subgroup analysis.

**Conclusion:**

Pretreatment GPS could serve as a simple and reliable prognosis predictor for NSCLC. More well-designed studies that consider GPS as a stratification factor are warranted.

## Background

For many decades, lung cancer has been the most common cancer and the leading cause of cancer death worldwide (Ferlay et al. 2010). Non-small cell lung cancer (NSCLC) accounts for approximately 80 % of lung cancer. Surgery, chemotherapy and radiotherapy are three primary treatment modalities for NSCLC. The current staging system, namely the TNM staging system, proposed by American Joint Committee on Cancer (AJCC) is widely used in clinical practice. However, the current TNM staging system is inadequate to predict prognosis. Prognosis differs in most cases, even for patients in the same stage. For example, approximately 30 % of early-stage patients experienced tumor recurrence and a poor prognosis although adequate radical treatment was administered. In recent decades, systematic inflammation has been thought to play a crucial role in tumorigenesis and serve as a hallmark of cancer (Hanahan and Weinberg 2011). The Glasgow prognostic score (GPS) is an inflammation-based scoring system that is evaluated by two serum indicators: C-reactive protein (CRP) as an indicator of systematic inflammatory response and albumin, which reflects nutritional status (Bremnes et al. 2011; Liao et al. 2014; Forrest et al. 2004; Leung et al. 2012; Pinato et al. 2014). Several studies have reported the correlation between GPS and NSCLC patients; however, due to differences in the inclusion criteria of NSCLC patients and limited sample sizes undermining its role, its significance in patients with NSCLC has not been fully studied. Considering that the GPS is currently widely studied for different types of cancers, including NSCLC, a systemic comparison to further clarify its clinical utility is needed. Therefore, this study aimed to explore the prognostic value of GPS in NSCLC using a quantitative meta-analysis combining all available evidence from retrospective studies.

## Methods

### Publication search

A systematic search was performed in PubMed, Web of Science, and the Cochrane Library from January 1, 1966 to August 31, 2015. The search strategy used both MeSH terms and free-text words to increase sensitivity. The following search terms were used: “inflammation-based score”, “Glasgow prognostic score”, “GPS”, and “non-small cell lung cancer”.

### Inclusion and exclusion criteria

The following inclusion criteria were used: (1) articles investigating the relation of GPS and prognosis for NSCLC patients; (2) serum was collected before surgery or other treatments; (3) patients were grouped according to the GPS; (4) GPS 0/1/2 was defined as none/only one/both elevated CRP (>10 mg/L) and hypoalbuminemia (<35 g/L). The following exclusion criteria were used: (1) letters, editorials, expert opinions, case reports and reviews; (2) studies without usable data; (3) duplicate publications.

### Data extraction

Two investigators independently extracted data from the eligible studies, and disagreements were resolved by discussion with a third investigator. For each study, the following information was recorded: first author, publication year, country, sample size, TNM stage, therapy, and overall survival (OS).

### Statistical analysis

The hazard ratios (HRs) were extracted as described in our previous publication (Zhu et al. 2015). The procedure is briefly outlined: (1) obtain the HRs directly from the publication; HRs from multivariate analysis are an option when HRs from univariate analysis were also available; (2) estimate the HRs from O-E statistic and variance; (3) calculate the HRs from data, including the number of patients and events at risk in each group, and the log-rank statistic or the *p* value; (4) retrieve HRs from Kaplan–Meier Curves by extracting several survival rates at specified times from the curves. Statistical analyses of HRs for OS were calculated by Review Manager Version 5.3 (Revman, the Cochrane Collaboration, Oxford, England). The heterogeneity of the data was evaluated by Chi square *Q* test and *I*^2^ statistic. For the *Q* test, a *p* value <0.05 indicated significant heterogeneity; for the *I*^2^ statistics, an *I*^2^ value greater than 50 % was considered significant heterogeneity. Statistical significance was defined as a *p* value <0.05.

## Results

### Characteristics of included studies

**Fig. 1:**
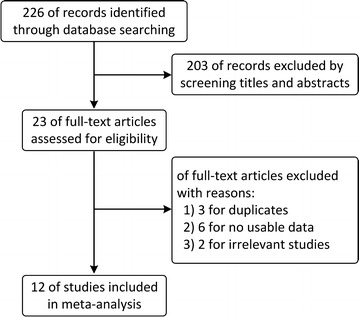
The flow diagram of this meta-analysis

As shown in Fig. 1, the electronic search yielded 226 records. After screening titles and abstracts, 23 full-text articles were assessed for eligibility. Finally, a total of 12 articles met the inclusion criteria and were included in this meta-analysis (Forrest et al. 2004; Leung et al. 2012; Pinato et al. 2014; Miyazaki et al. 2015; Kishi et al. 2015; Kawashima et al. 2015; Jiang and Lu 2015; Grose et al. 2015; Tomita et al. 2014; MacKenzie et al. 2014; Rinehart et al. 2013; Meek et al. 2010). Among these 12 articles, six were retrospective, and six were prospective studies. Six studies were from United Kingdom, four from Japan, one from China, and one from America. Details are summarized in Table 1.

**Table 1:** Characteristics of studies in this meta-analysis

Study name	Region	Study type	Sample size				Stage	Therapy
			Total	GPS 0	GPS 1	GPS 2		
Miyazaki et al. (2015)	Japan	Retrospective	97	65	32		I	Surg
Kishi et al. (2015)	Japan	Retrospective	165	100	65		I	SBRT
Kawashima et al. (2015)	Japan	Retrospective	1043	897	107	39	I–III	Surg
Jiang and Lu (2015)	China	Prospective	138	95	32	11	III–IV	Chemo
Grose et al. (2015)	UK	Prospective	120	50	49	21	I–III	Surg/RT/Chemo
Tomita et al. (2014)	Japan	Retrospective	312	264	31	17	I–III	Surg
Pinato et al. (2014)	UK	Prospective	220	131	39	29	I–III	Surg
MacKenzie et al. (2014)	UK	Retrospective	97	–	–	–	I–III	Surgery/RT
Rinehart et al. (2013)	USA	Prospective	51	9	32	10	IV	Chemo
Meek et al. (2010)	UK	Retrospective	56	19	31	6	Inoperable II–IV	RT/Chemo
Forrest et al. (2004)	UK	Prospective	109	27	69	13	Inoperable III–IV	Chemo
Leung et al. (2012)	UK	Prospective	261	59	163	39	Inoperable III–IV	RT/Chemo

*Surg* surgery, *SBRT* stereotactic body radiation therapy, *RT* radiotherapy, *Chemo* chemotherapy

### Association between GPS and OS

**Fig. 2:**
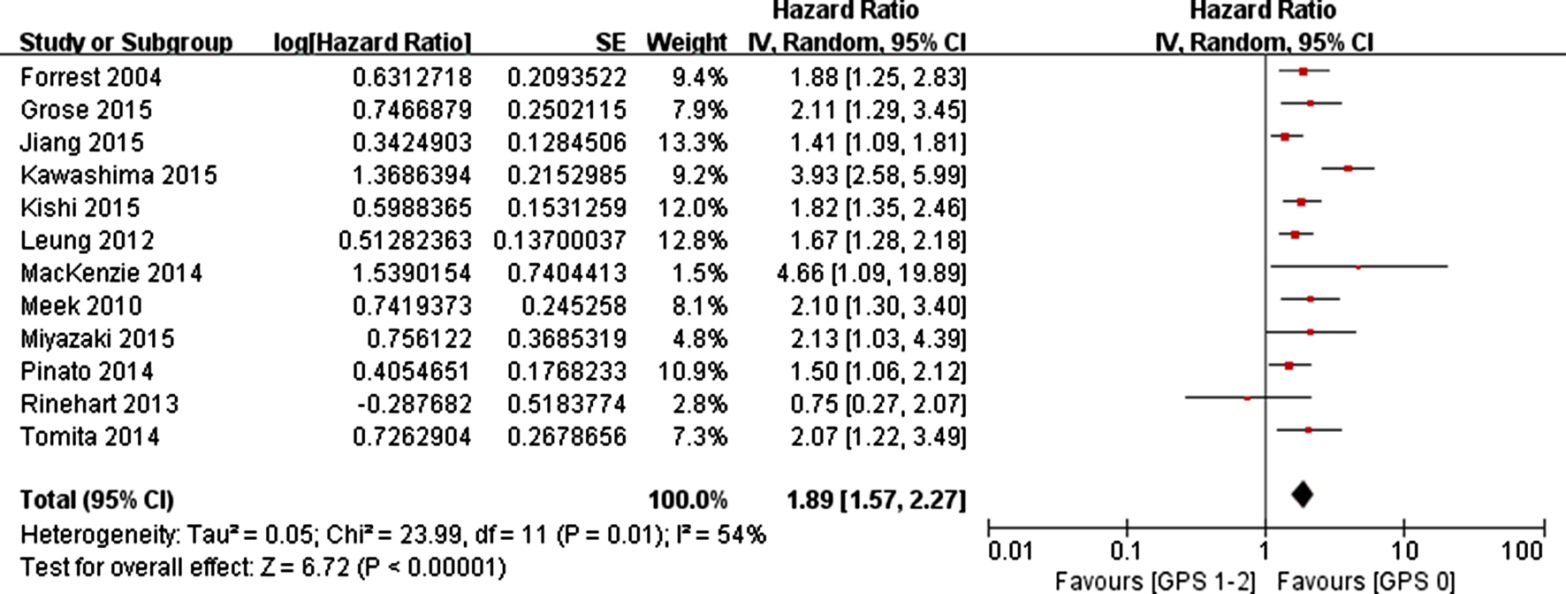
The pooled HRs of OS for GPS 0 versus GPS 1–2

In total, 12 studies of 2669 patients compared OS between GPS 0 and GPS 1–2 groups. The random-effects model was adopted as the significant heterogeneity (*I*^2^ = 54 %, *p* = 0.01). Analysis revealed a pooled HR of 1.89 with 95 % CI 1.57–2.27 (*p* < 0.001) (Fig. 2). Compared with GPS 0 group, patients in GPS 1–2 group had a significantly poor OS, suggesting pretreatment GPS had the ability to predict prognosis.

**Fig. 3:**
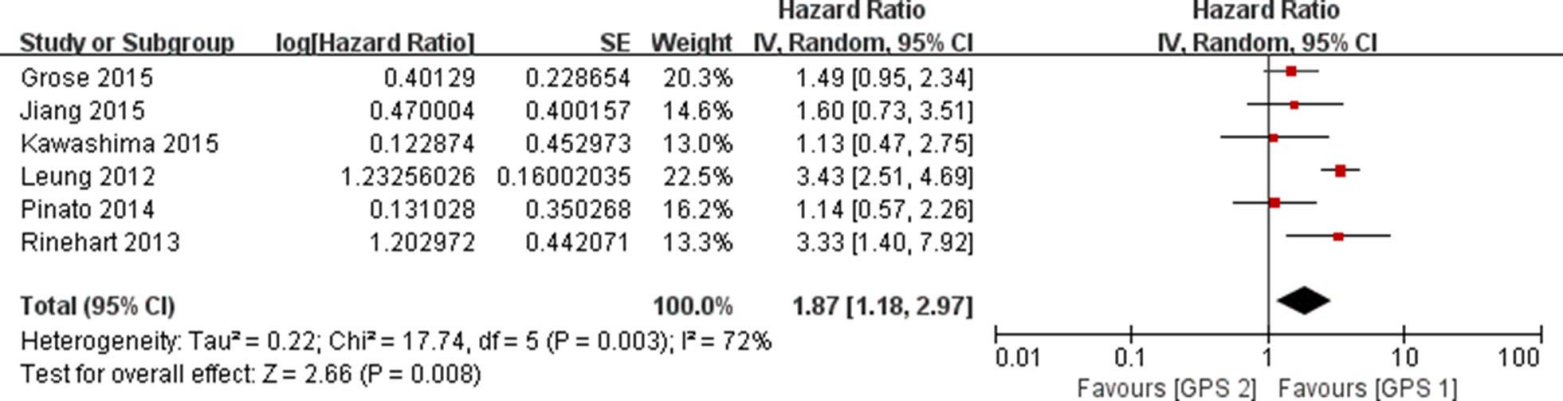
The pooled HRs of OS for GPS 1 versus GPS 2

Six studies had sufficient data to calculate HRs of OS between GPS 1 and GPS 2 groups. Because significant heterogeneity existed among the studies (*I*^2^ = 72 %, *p* = 0.003), the random-effects model was adopted. Analysis revealed a pooled HR of 1.87 (95 % CI 1.18–2.97, *p* = 0.008) (Fig. 3). GPS 1 group had a significantly prolonged OS compared with GPS 2 group, indicating an increased risk of patients with both elevated CRP and hypoalbuminemia.

### Subgroup analysis

As significant heterogeneity was noted across the studies, we further investigated potential sources of heterogeneity by subgroup analysis.

We first explored the impact of study type (retrospective vs. prospective) on heterogeneity. Six retrospective studies had a pooled HR (GPS 0 vs. GPS 1–2) of 2.38 (95 % CI 1.76, 3.20) with small heterogeneity (*I*^2^ = 49 %, *p* = 0.08), and six prospective studies had a pooled HR (GPS 0 vs. GPS 1–2) of 1.58 (95 % CI 1.37, 1.83) with small heterogeneity (*I*^2^ = 3 %, *p* = 0.40). Next, we explored the influence of disease stage on heterogeneity. Five studies including stage IV or inoperable patients had a pooled HR (GPS 0 vs. GPS 1–2) of 1.61 (95 % CI 1.34, 1.94) with small heterogeneity (*I*^2^ = 21 %, *p* = 0.28). These results indicate that pretreatment GPS might predict prognosis well.

## Discussion

The current study is a quantitative meta-analysis evaluating the prognostic value of GPS in patients with NSCLC. The pooled estimates of 12 studies involving 2669 patients indicated that patients with elevated GPS were predisposed to exhibit inferior survival outcome (HR 1.87: 1.18–2.97, *p* = 0.008). The significant relationship between GPS and OS was assessed in all the subgroup analyses stratified by the study type (retrospective vs. prospective) and different stages (early stage vs. stage IV or inoperable). The results indicate that GPS can be a practical indictor to predict NSCLC patient prognosis.

Inflammation plays an important role in tumor occurrence and development (O’Callaghan et al. 2010; Tauler and Mulshine 2009). GPS, which is an inflammation-based score combining serum CRP and albumin, was first proposed by Forrest et al. (2003). Subsequently, its clinical significance has been evaluated in various tumor types, including hepatocellular carcinoma, gastric cancer, prostate cancer, and colorectal cancer. CRP, a non-specific acute reactant protein of inflammation, is synthesized in hepatocytes and in response to release of cytokines, such as interleukin 6 release by monocytes and other immune cells under infection, tissue necrosis, and inflammatory disease (Pepys and Hirschfield 2003). As a component of the inflammatory response of the immune system, CRP exerts important role in the tumor-host interaction (Ballou and Lozanski 1992; Cermak et al. 1993), and elevated CRP is associated with impaired T lymphocytic response within the tumor (Du Clos and Mold 2004). A meta-analysis conducted by Jing et al. (2015) indicated that elevated CRP could predict poor 5-year OS rates (RR = 2.15, 95 % CI 1.78–2.59) and 5-year disease-specific survival rates (RR = 2.12, 95 % CI 1.56–2.88). Serum albumin is an important marker of nutritional status, and hypoalbuminemia is correlated with cachexia (Evans et al. 2008). Mori et al (2015). found that the prognostic nutritional index was an independent prognostic factor for completely resected NSCLC. Another study indicated that pretreatment serum albumin was an independent prognostic factor for Stage IIIB NSCLC that is associated with the response rate to first-line therapy and survival rates (Tanriverdi et al. 2015). Additionally, hypoalbuminemia is associated with treatment-induced toxicity or complications (Ataseven et al. 2015; Arrieta et al. 2010), which also may portend the poor prognosis to a certain extent.

Currently, accurate staging of the tumor is of vital importance in making a clinical decision of NSCLC. The International Association for the study of Lung Cancer (IASLC) periodically proposes revisions to the TNM staging system, and increasing data indicates information used to indicate tumor stage is inadequate (McMillan 2008). Performance status (PS) is an effective complement for tumor stage, partly reflecting patient’s tolerance to treatment. For patients with poor PS, chemotherapy or radiotherapy are typically not applicable. Several large-scale studies indicate that PS is an independent prognostic factor for OS in NSCLC (Kawaguchi et al. 2010; Sculier et al. 2008; Chansky et al. 2009). However, PS is recognized to be subjective, and its reliability in predicting outcome is compromised (Ando et al. 2001). By contrast, as an objective predictor, GPS is easier to measure, routinely available and well standardized. Increasing evidence indicates that GPS has independent prognostic value in cancer patients (McMillan 2008, 2013). A study of Forrest et al. (2004) also indicated that the prognostic value of the GPS is independent of tumor stage in NSCLC with an HR of 1.88 (95 % CI 1.25–2.84). Our data indicate that elevated GPS scores correlate with inferior survival, suggesting that the use of anti-inflammatory agents and the reinforcement of nutrition possibly may benefit the overall survival.

Nevertheless, there are several limitations in the study. The comparison between GPS 0 and GPS 1 was not performed as the relevant data were not reported in most of the included studies. Different treatment settings in studies also inevitably increased the heterogeneity. In addition, the large I^2^ value (54 and 72 %) reflected this dilemma and would reduce the reliability of interpreting the results. Some studies incorporating patients with small cell lung cancer were not included in our analysis (Grose et al. 2015; Gioulbasanis et al. 2012). Given that the number of patients included in each study for each meta-analysis was relative small and half of the included articles were retrospective studies that could generate bias by variation, a large scale prospective study that uses GPS as a stratification factor is required to reduce the bias in future.

In conclusion, as an easily obtained and reliable inflammatory index, GPS is a promising prognostic indicator in patients with NSCLC. More well-designed prospective studies are needed to promote the use of GPS in the routine clinical practice as a complementary prognostic factor for the current TNM staging system.
